# Hurdles for Phage Therapy to Become a Reality—An Editorial Comment

**DOI:** 10.3390/v11060557

**Published:** 2019-06-17

**Authors:** Harald Brüssow

**Affiliations:** KU Leuven, Group of Gene Technology, 3001 Leuven, Belgium; haraldbruessow@yahoo.com

This special issue of Viruses asks experts in the field about “Hurdles to phage therapy (PT) to become a reality”. Their answers came as reviews, perspectives and opinions, along with a number of research papers. No singular hurdle was identified by the authors. According to the specialization of the contacted scientists, various different hurdles or gaps in knowledge impeding progress with PT were described. Collectively, the analyses give, however, a valuable description of the status quo and hopefully provide some direction for future fundamental and clinical research in PT. In view of the grim specter of a possible return to a pre-antibiotic era for a number of bacterial infections, exploring alternatives or adjuncts to antibiotics are of high public health importance and need no further justification. PT is without doubt an interesting approach to the antibiotic resistance problem and merits intensified research to get out of the fruitless confrontation between enthusiasm from the East and lingering Western skepticism.

## 1. Overview on the Contributions to This Issue

In this special issue, I invited a wide range of authors covering a science journalist who is author of a well-documented book on the history of PT [1] and a representative from a non-governmental organization [2], representatives of industry and opinion leaders in academic PT research and its clinical and agronomical application. Societal awareness of the problem is necessary to assure sufficient political support, which is needed to finance the development of phage products and costly clinical trials for the regulatory acceptance of PT. In my opinion, the currently available evidence of PT seen through clinical trials is not yet a sufficiently strong incentive for the private sector to invest heavily in this field. It is therefore likely that the public sector needs to take the lead to prove the value of the PT approach. This is not an unfair request, since exploring the potential of alternative antimicrobial agents is a task of the public health sector in view of the challenge of untreatable bacterial infections, which might in the near future dwarf past challenges, even that of the HIV epidemic. Once the scientific and clinical evidence is published for PT, it is likely that the private sector will follow with more investments.

Patent and regulatory issues still cause some hesitation in the private sector. Official organizations such as the World health Organization (WHO), European Medicines Agency (EMA) and the US Food and drug Administration (FDA) did not want to define their position towards regulatory aspects of PT in this special issue. This resulted in an over-representation of national, particularly Belgian, personalized medicine approaches with two contributions from the military hospital in Brussels, where the magistral phage approach was developed [3]. Large pharmaceutical industries have not shown much interest in the PT approach so far. There might be a number of reasons for this situation. On one side, there is no economic incentive to develop new antibiotics and even less developing non-antibiotic alternatives to their current antibiotic business. In addition, the classical pharmaceutical industry deals with small chemical drugs—or at most proteins that are molecularly well-defined—while phages represent large, replication-competent, biological material that is subject to biological variation and evolution. Thus, defining the composition of a phage product is not trivial and several contributions to this issue address this problem [4,5,6,7,8]. The pharmacokinetic properties of phages raise issues unknown to standard pharmacology approaches.

The fact that one of the few randomized controlled trials (RCT) with PT conducted by the private sector was organized by a food company (Nestlé, Vevey, Switzerland) should not be a surprise. Fermentation using bacterial starter cultures is used in various food production processes. These processes are always threatened by phage attacks, necessitating substantial phage research in the food industry, particularly in the field of phage-resistance. Food companies have therefore maintained active phage research groups. With the extension of several food companies into the nutrition and health area, the human microbiome research has come into focus, and with it again, phages as modulators of the bacterial microbiome. Using phages to correct microbiome dysbiosis is a potentially interesting application beyond just targeting single bacterial pathogens.

Fauconnier [9] proposes in his contribution an adapted approach to the regulation of PT. This point of view was not at all shared by one reviewer of the paper, who adamantly claimed that the current drug legislation both in Europe and North America is sufficient for PT introduction and that no alternatives exist to RCT demonstrating safety and efficacy if PT wants to see the market place. These two opposing views describe an unsettled controversy, although I personally think that both approaches are not mutually exclusive. A personalized medicine approach with phages, as currently under development in Belgium, will fulfill a pioneer function for PT in Western countries. Once sufficient efficacy data has been accumulated with that approach and with numbers of untreatable bacterial infections going into the several hundred of thousands, personalized medicine approaches will no longer be practical and phages would need to be developed as common drugs, provided that they show efficacy in RCT. The paper of Philipson et al. [7] describes how phages can be produced to FDA standards. Other frequently quoted issues hampering the introduction of PT, such as the difficulty of patenting approaches [10] or the problem of rapid phage-resistance development [11,12,13] are discussed and found to be less critical than commonly assumed. Oechslin even raises the possibility to explore Darwinian medicinal approaches, where phage treatment can induce virulence attenuation or reestablish antibiotic sensitivity [12]. Casey et al. argue that part of the clinical problems with PT can already be settled by careful selection with in vitro tests better suited for reflecting real life situations [14]. However, other contributions point to complications of PT that can only be assessed in a realistic in vivo context reflecting ecological [15] or evolutionary constraints [16] encountered at organismal or even population levels. I agree strongly with the notion that the lack of detailed in vivo knowledge of phages currently limits our capacity to design and eventually assure successful clinical trials. This in vitro orientation of phage research has historical reasons: phages were investigated by scientists under the perspective of the reductionist principle, which led to the molecular biology revolution [17]. This situation is likely to change with phages now returning on the scene when microbiome research has discovered the importance of phages in regulating microbial ecosystems as different as the oceans and the gut.

This special issue solicited insights from major stakeholders in the medical PT field, including a lead scientist from an industrial group that conducted the only successful RCT in PT [18], or the Polish [19] and Georgian PT centers; the latter with a contribution demonstrating how they select a therapeutic phage against a specific emerging pathogen in the field [20]. Compassionate phage use in the USA [21], in France [22] and with the Belgian Magistral Phage preparation [23] are described. Phage use in food production is reviewed by scientists from Intralytics [24] and phage in the service of agriculture is described by Svircev et al [25].

Considering that PT is a wide field, some subjects are only represented with a single paper: van Belleghem et al. [26] explore the impact of phage on the immune system, Thiry et al. [27] the use of a simple animal model for screening large numbers of phages for simplified in vivo phenotypes; Roy et al. [28] explore an interesting phage production system; Lin et al. [29] investigate the use of phage enzymes for infection treatment. However, some features important for the assessment of PT are missing, such as a failure analysis of the Phagoburn clinical trial [30] that had been supported by a grant from the European Community. A thorough microbiological work-up of failed RCTs are of substantial importance for future PT trial planning, such as that sponsored by the German government, where suitable phages against lower respiratory tract infections will be selected at the Leibniz Institute, produced to GMP standards at the Fraunhofer Institute and clinically tested at the Charité hospital as described by Wienhold et al. [31] in this issue.

## 2. Failure Analysis of the Bangladesh Diarrhea PT Trial

Since I was actively involved in a failed PT trial, the Nestlé diarrhea trial in Bangladesh [32], I will here summarize my personal evaluation of hurdles to phage therapy.

Perhaps it is best to start with what was not a hurdle in that RCT. Two aqueous phage products, a commercial Russian phage cocktail [33] and a phage cocktail specifically produced for this trial at the Nestlé Research Center [34] were tested. While maintained for the RCT over several years under refrigeration conditions, no decline in phage titer was seen [35], in contrast to initial experiences in the Phagoburn trial. The International Center for Diarrhoeal Disease Research in Bangladesh (icddr,b), the world’s leading diarrhea research hospital, has a straightforward review process for clinical protocols consisting of four steps: in-house evaluation, external review, a research, followed by an ethical committee in Bangladesh. In fact, it was more difficult to get the export permit for phages from Russia than to get to their import permit into Bangladesh, once the protocol was approved by the ethical committee. Since oral phage use was planned, we only needed a food-grade phage preparation. Establishing a RCT for PT was not a difficulty, provided that all patients got the most efficient standard treatment consisting of oral rehydration solution supplemented with zinc. Since zinc already has a shortening effect on diarrhea duration, PT had to show an advantage over zinc treatment alone; this is a fair request in view of the low cost and risk of zinc supplementation. The start of the efficacy trial was delayed because the icddr,b clinicians asked for supplementary safety tests in healthy subjects of gradually decreasing age from Bangladesh [36] in addition to a safety test in adult Swiss healthy subjects [37]. Interestingly, external reviewers argued that healthy subjects would carry the risk of phage exposure without the possible therapeutic benefit of phage. Phage has been applied to many healthy subjects in Bangladesh and elsewhere without observing adverse events. As phage is not toxic as virion, but only when lysing the bacterial host during infection and releasing toxic bacterial products, the ethical committee in Bangladesh has subsequently also approved nasal application of commercial staphylococcal phage products from the Eliava Institute in Georgia [38]. The quality of clinical follow-up is very good at icddr,b, as documented by many influential publications coming from this research hospital. There is thus no objective hurdle to conduct RCTs with PT in Bangladesh to obtain scientific evidence for PT efficacy.

Now to the hurdles: there are indeed physico-chemical hurdles to phage use. In vitro experiments suggested heavy phage loss during simulated gastric passage conditions [34]. The ethical committee in Bangladesh did not allow buffering of gastric acidity in patients for concern of increased nosocomial infection risk in a diarrhea hospital with heavy pathogen load. We therefore probably lost a substantial amount of the orally applied phages in gastric passage. There are solutions to this problem (increasing the oral dose, microencapsulation), but we did not anticipate this difficulty since we had observed good oral phage transit in adult Swiss volunteers [37]. Since children and adults from developing countries produce less stomach acidity (hypochlorhydria) than Western adults [39], we anticipated an even better gut transit, which was not the case, therefore indicating limitations in our knowledge about the pharmacokinetics of oral phage products in subjects of the developing world. Apparently, more attention has to be paid to galenic preparations of phages to get phages at sufficient titers to the site of action of the targeted bacterial pathogen.

Laboratory analysis of the clinical samples also identified other factors that prevented clinical efficacy of the oral phages. As acute *Escherichia coli* diarrhea was the target for PT in this trial, phage treatment was started after rapid exclusion of non-*E. coli* diarrhea (rotavirus, cholera, shigellosis). However, further analysis revealed that only half of the enrolled cases showed a confirmed *E. coli* infection [32]. Many pathogens are involved in diarrhea, and a given pathogen might represent only a moderate share of all acute diarrhea cases. This observation is not restricted to diarrhea, but also applies to pneumonia, the major killer of children in developing countries. Under this condition, only a fraction of the treated patients would profit from a treatment with a phage preparation targeting a single pathogen. This problem can of course be addressed by using complex phage cocktails like Intestiphage preparations from Russia (Microgen) or Georgia (Eliava) containing phages against many enteropathogen species. Even then, two problems remained: first, even in confirmed cases of *E. coli* infection, *E. coli* did not represent the dominant bacterium in the stool [32]. Acute diarrhea cases showed a dominance of intestinal streptococci independent of their etiology in the stool [40], and this dysbiosis normalized with recovery from diarrhea. Diarrhea output correlated with streptococcal, but not *E. coli* stool abundance. In fact, the concentration of fecal pathogenic *E. coli* was near or below the replication threshold determined for T4-like coliphages to maintain an infection chain in the laboratory [32]. Second, acute diarrhea in children from developing countries is typically a polymicrobial infection [41], and this was also our observation. In addition, several *E. coli* pathotypes showed a low pathogenicity index in epidemiological surveys of children from developing countries [42], raising doubts about their role as pathogens. Due to this complexity, acute diarrhea is unlikely to represent a suitable target for PT. The problem is further compounded by the genetic variability of *E. coli*. Even with phage cocktails containing 10 phage strains, we achieved only about 50 per cent coverage (i.e., in vitro lysis) of the fecal *E. coli* isolates from the patients [32,43]. When including more phage strains, we encountered interference problems, where the cocktail showed less coverage than the sum of the individual phages.

## 3. Recommendations

The take home lessons from our PT experience are thus: successful PT trials are more likely with infections where:

(1) The disease-causing role of the bacterial pathogen is clearly established. Do not rely on textbook knowledge and confirm the role of the pathogen in your targeted patient population.
(2) Polymicrobial infections should be avoided or addressed with a multi-pronged approach.
(3) The pathogen is present with a sufficiently elevated concentration to allow productive phage infection chains to occur in the patient.
(4) Suitable phages are available to cover the genetic diversity of the pathogen.

Suitable phages are not always at hand. For example, when researchers screened a collection containing more than 10,000 mycobacteriophages (the largest collection of characterized phages directed against a single bacterial genus) for the treatment of two cystic fibrosis patients infected with *Mycobacterium abscessus*, they found only one lytic phage for one patient [44]. By genetic engineering they could transform a second temperate phage into a suitable lytic phage by deletion of the phage repressor. For two other phages, suitable host range mutants containing spontaneous point mutations were selected. The good news is that a cocktail of three phages, containing a genetically-engineered phage, was approved for clinical use and rescued one patient. This point proves that even a genetically modified phage was approved for patient use in Europe and this fact extends the possibilities offered to PT substantially. However, the bad news was that for the other patient, infected with another *M. abscessus* strain, no suitable phage could be found and the patient died. In contrast, some phage types have an extremely wide host range on *S. aureus*, including methicillin-resistant and to a lesser extent vancomycin-resistant strains; however, they also infect *S. epidermidis*, which represents a potential collateral damage on a skin commensal in skin application.

RCT of PT are more difficult to organize with acute rather than with chronic infections, since short disease durations need an early phage intervention frequently before the microbiological diagnosis becomes available, resulting in the enrolment of many uninformative patients. In contrast, prevention of acute diarrhea might be more attractive when the epidemiological situation is clear: for example, in case of prophylactic phage treatment of contact persons from cholera patients or outbreaks of cholera epidemics in refugee camps. In fact, the large successful prevention clinical trial of Shigella diarrhea conducted by the Eliava Institute in 1963 supports this point [45]. However, prevention trials depend on a careful follow-up causing logistic problems, thus making them frequently more costly than treatment trials of PT.

An additional hurdle is the fact that the targeted pathogen must be accessible to the applied phage. While oral phage application seems, at first view, an appropriate way to treat a gastro-intestinal infection, there are barriers beyond phage inactivation in the stomach. Gut peristalsis is accelerated in diarrhea and it becomes questionable if oral phage has long enough contact times to infect a pathogen like *Vibrio cholerae* [46]. Furthermore, it is not clear where the enteropathogen is actually located; is it in the lumen, in the mucus layer or epithelium-associated? Enteropathogens display a variety of virulence genes that allow them to penetrate the mucus layer and to adhere to gut epithelia. Some phages display depolymerase enzymes at their tail fibers, which allow penetration of bacterial capsular layers and sometimes bacterial biofilms. It is less clear whether phages are able to follow bacteria that adhere to the epithelia through the mucus layer. Mouse experiments showed that an in vitro fully-susceptible bacterial host could escape infection in the gut without developing genetically determined phage resistance. In this case, phage replicated in vivo only on a subpopulation of the host bacteria [47,48,49]. We still do not know enough about the physiological differentiation of bacteria in the mammalian gut. While clinical sampling is principally possible to study phage-pathogen interactions in at least some accessible gut segments of patients, the procedures are invasive and ethical committees will not allow invasive sampling that is not clinically indicated. It is thus preferable to target infections on more accessible body sites in future PT trials where sampling is easier than the gut. Purulent bacterial skin infections with *Staphylococcus aureus* or *Streptococcus pyogenes* come to mind.

Microbiome studies on the skin have demonstrated a substantial depth differentiation for bacterial colonization of the skin. Even in such “easy” sites for topical phage application like the skin, it remains to be shown in what epidermal cell layer the pathogen resides and whether phage can reach them. In fact, phages are commonly selected for vigorous in vitro planktonic growth on their target bacterium maintained under optimal nutrition. However, these are idealized laboratory conditions. In vivo, many bacteria grow very slowly in biofilms or in mucus layers. One might therefore ask whether we should not select phages for PT that are able to infect bacteria in biofilms or under simulated slow in vivo growth conditions. Complex biofilms consisting of different bacterial species are difficult to realize in the laboratory and not suitable for testing large numbers of source material containing phages (but see Thiry et al. [27] in this issue). Some in vivo properties can be predicted from in vitro observations (see Casey et al. [14] in this issue). For example, T4-like coliphages only replicate on exponentially growing *E. coli* cells, while T7-like phages replicate also on *E. coli* in stationary phase [47].

## 4. Outlook

Clearly, we need more ecophysiological data on in vivo phage-bacterium interaction in relevant animal models to select suitable phages for clinical application. As argued by Torres-Barceló [16] in this issue, evolutionary thinking should be included in this reasoning. A phage that kills off its host bacterium, present at low concentrations, wipes out its growth substrate and is unlikely to be maintained in evolution. Based on theoretical reasoning, phages should be active on expanding bacterial populations that shift the ecosystem to a state dominated by one or few bacteria. Phages might therefore play a positive role in ecology by maintaining bacterial genetic diversity in the environment [50]. This argument meets the threshold concept for phage replication and might suggest that PT could be more effective in fighting microbial dysbiosis due to an outgrowth of undesired bacteria as in antibiotic-associated diarrhea than against pathogens which mediate clinical effects while present in low numbers. If low-level food contaminants were to be eliminated, very high phage titers were needed to achieve enzymatic “lysis from without” rather than by phage replication.

From these arguments, one might conclude that we need more fundamental knowledge on phage-bacterium interaction in pertinent animal models before successful clinical application can be envisioned for PT. A possible short-cut to successful PT could be the careful evaluation of past personal experience [18,22], compassionate phage use [21], systematic evaluations of case reports [19] and patient follow-up with magistral phage preparation [3,23], all discussed in this issue. Case reports combining clinical observation with state-of-the-art laboratory investigation of in situ phage–bacterium–host interactions might pave the way to successful RCT with PT. We should avoid to target infections for PT according to the scientific background of the research group and their “favorite infection”. The EMA and FDA have already called conferences for stakeholders of PT, without much concrete recommendations. Perhaps public health authorities should convene a consensus finding conference for the best target of PT for a RCT sponsored by the Horizon 2020 calls of the EU.
